# Salicylate induces epithelial actin reorganization via activation of the AMP-activated protein kinase and promotes wound healing and contraction in mice

**DOI:** 10.1038/s41598-024-67266-5

**Published:** 2024-07-16

**Authors:** Kento Takaya, Keisuke Okabe, Shigeki Sakai, Noriko Aramaki-Hattori, Toru Asou, Kazuo Kishi

**Affiliations:** Department of Plastic and Reconstructive Surgery, Keio University School of Medicine, 35 Shinanomachi, Shinjuku-Ku, Tokyo, 160-8582 Japan

**Keywords:** Molecular medicine, Disease model, Drug development

## Abstract

Wounds that occur in adults form scars due to fibrosis, whereas those in embryos regenerate. If wound healing in embryos is mimicked in adults, scarring can be reduced. We found that mouse fetuses could regenerate tissues up to embryonic day (E) 13, but visible scars remained thereafter. This regeneration pattern requires actin cable formation at the epithelial wound margin via activation of adenosine monophosphate (AMP)-activated protein kinase (AMPK). Here, we investigated whether the AMPK-activating effect of salicylate, an anti-inflammatory drug, promotes regenerative wound healing. Salicylate administration resulted in actin cable formation and complete wound regeneration in E14 fetuses, in which scarring should have normally occurred, and promoted contraction of the panniculus carnosus muscle, resulting in complete wound regeneration. In vitro, salicylate further induced actin remodeling in mouse epidermal keratinocytes in a manner dependent on cell and substrate target-specific AMPK activation and subsequent regulation of Rac1 signaling. Furthermore, salicylate promoted epithelialization, enhanced panniculus carnosus muscle contraction, and inhibited scar formation in adult mice. Administration of salicylates to wounds immediately after injury may be a novel method for preventing scarring by promoting a wound healing pattern similar to that of embryonic wounds.

## Introduction

When wounds occur in adult mammalian skin, the tissue does not regenerate; instead, it is replaced by fibrotic scar tissue, leaving visible scars that reduce the patient’s quality of life^1^. Our understanding of the mechanisms that prevent or promote skin regeneration is limited, and no effective treatment exists to fully regenerate the structure and function of the skin. In contrast, fetal wounds heal through regeneration without scarring^2–4^. Fetal regeneration has been demonstrated in a variety of tissues and may serve as a model for tissue regeneration.

One mechanism that plays an important role in fetal skin regeneration is the formation of actin filaments at wound margins. Initially proposed by Martin et al., this concept hypothesizes that an actin cable extends from cell to cell at the anterior margin of the healing embryonic epithelium, forming a ring around the wound that acts as a contractile “drawstring” and facilitates wound closure^5,6^. Actin cable formation does not occur during adult wound healing; instead, keratinocytes migrate to the exposed connective tissue using filopodia to close the wound^7^.

In a previous study, we reported that the timing of the shift between complete skin regeneration and non-regeneration during mouse development coincided with the presence or absence of actin cable formation^8^. During this time, the three-dimensional structure of the skin was completely regenerated from a full-layer skin incision created before embryonic day (E) 13. Actin cables were observed during the entire wound-healing process, and the epidermis and dermis maintained their positional relationships. However, the skin chyme did not regenerate in the wounds after E14. The regulation of microtubule and actin filament behavior by adenosine monophosphate (AMP)-activated protein kinase (AMPK) is important for regulating actin cable formation and cell migration^9^. In particular, the manipulation of AMPK activity is necessary to reproduce the pattern of skin regeneration by actin cable formation in the embryonic form. Reports on AMPK activation in wound healing have presented varying results. For example, AMPK activation in endothelial cells has been shown to cause several biological effects that promote vascular homeostasis, including suppression of hyperglycemia-induced generation of reactive oxygen species, reduction of lipotoxicity induced by free fatty acids, and protection against apoptosis^10,11^. Several activators of AMPK have been reported^12^, among which we found in our previous study that compound 13 causes partial wound regeneration by altering actin dynamics during fetal mouse wound healing via a pathway involving AMPK activation. However, treatment with this drug causes a significant reduction in epidermal cell migration and may prolong wound epithelialization in adults^13^.

Salicylate is one of the oldest medicinal compounds known to humans and has been widely used to reduce fever, pain, and inflammation^14^. Various potential mechanisms of action of salicylate have been proposed, including the inhibition of nuclear factor kappa B (NF-κB) activity via IκB kinase β (IKKβ) or the regulation of histone acetylation by inhibiting CBP/p300^15^. However, the multifaceted effects of salicylate on various cell types remain unclear. Recently, a novel action of salicylate was discovered, with a recent study showing that it activates AMPK by binding to the A-769662 drug-binding site on the AMPK β1 subunit^16^. Through this mechanism, salicylate reportedly improves atherosclerosis and fatty liver^16,17^. However, the role of salicylate in wound healing remains unclear. Aspirin, a prodrug of salicylate, improves diabetic ulcers by ameliorating chronic inflammation; however, this process is not mediated by AMPK activity^18^.

We hypothesized that salicylate administration to acute wounds alters actin dynamics via AMPK activation, thereby promoting skin regeneration and healing. Using an originally developed mouse fetal wound healing model, we administered salicylate to the wounds of either fetuses or adult mice during the period in which the skin could not regenerate and observed its effects on wound healing.

## Materials and methods

The research protocol was reviewed and approved by the Animal Experimentation Committee of Keio University School of Medicine (approval number: 20170914). All experiments were performed in accordance with the institutional guidelines for animal experiments at Keio University. This study was conducted in accordance with the Animal Research: Reporting of In Vivo Experiments (ARRIVE) guidelines.

### Fetal wounding procedure

ICR mice were used as models for this experiment. All mice were obtained from Sankyo Laboratory Services (Tokyo, Japan). Vaginal plugs were checked twice daily. The day on which the plug was observed was designated as E0. The fetuses were injured at E13, E14, and E15. Surgery was performed on four pregnant mice at each time point, and wounds were created in at least four fetuses per pregnant mouse. The mothers were divided into a control group and salicylate-treated group. Pregnant mice were anesthetized with isoflurane, and the abdominal wall was incised to expose the uterus. The myometrium and amnion/yolk sacs were excised under an operating microscope. A full-layer incision approximately 2 mm in length was created in the lateral thoracic region of the fetus using surgical micro-scissors. At the time of wounding, 100 µL of PBS was administered to the control group, whereas the salicylate group received 100 µL of salicylate (0.1, 1, 10 mM, Sigma-Aldrich, St. Louis, MO, USA) dissolved in phosphate-buffered saline (PBS). When fetuses were retrieved 36, 48, or 72 h after surgery, the wound was labeled with 0.25% 1,1′-Dioctadecy-3,3,3′,3′-tetramethylindocarbocyanine perchlorate (DiI) dissolved in 1% ethanol in 10 µl of phosphate-buffered saline (PBS) to mark the wound area and for visualization. On E15, after wounding, the myometrium was sutured with 9–0 nylon, the uterus was returned to the abdominal cavity, and the abdomen was closed. The uterine relaxant ritodrine hydrochloride (Fujifilm Wako Pure Chemicals Co., Ltd., Osaka, Japan) was administered intraperitoneally at 1 μg/g body weight immediately prior to wound closure. The peritoneum and skin were then sutured using a 5–0 nylon thread. Maternal mice were euthanized by cervical dislocation, and fetuses were harvested 24, 48, and 72 h after injury. DiI fluorescence of the wounds was examined using a stereomicroscope (SZX16, Olympus Corporation, Tokyo, Japan) and an ultra-high-precision digital microscope (VHX-8000, Keyence Corporation, Osaka, Japan). The skin from the fetal wound area was harvested and fixed in 4% paraformaldehyde for 24 h. The fixed tissues were embedded in paraffin and stained. For immunostaining, the tissues were soaked in 20% sucrose/PBS after fixation, frozen, embedded in OCT compound (Sakura Finetek Japan Inc., Tokyo, Japan), and sliced into 7-µm sections.

### Adult mouse wounding procedure

Ten-week-old male ICR mice were anesthetized by isoflurane inhalation, and 8-mm dermopunches were made on the shaved back skin to create two 8-mm diameter full-layer wounds per mouse on both sides of the chest. The injured mice were divided into two groups (*n* = 8/group): the control group (PBS 100 µL) and the salicylate group (1 mM, 100 µL, dissolved in PBS). The wounds were treated daily with the reagent and covered with a film to avoid external irritation. Wounds were photographed weekly, and the wound area was calculated using ImageJ software. Healed skin was harvested 28 days after wounding for histological and molecular analyses.

### Cell scratch assay

Mouse epidermal keratinocytes PAM212 (Thermo Fisher Scientific, Waltham, MA, USA) and skin fibroblasts were established from the dermis of adult ICR mice via the explant method^8^ and passaged 5–7 afterwards were grown to confluency on plastic dishes. PAM212 cells were cultured in calcium-free Dulbecco’s modified Eagle’s medium (DMEM; Fujifilm Wako Pure Chemicals) supplemented with 10% heat-inactivated fetal bovine serum, 1 mM pyruvate, 2 mM glutamine, 1% penicillin/streptomycin, and 0.05 mM CaCl2. Fibroblasts were grown in low-glucose Dulbecco's modified Eagle’s medium supplemented with 1% penicillin/streptomycin and 10% fetal bovine serum. Before each assay, the cells were treated with mitomycin C (NAKARAI TESQUE, Inc., Kyoto, Japan) to exclude proliferative effects (10 µg/ml for 3 h at 37 °C). A 500-µm scratch was made using a pipette tip. Various concentrations of salicylate solution (0,0.1, 0.25, 0.5, 1, and 5 mM, each in 100-µL of PBS) were administered to the treated group, whereas the same volume of PBS (100-µL) was added to the control group. After 48 h, the cells were observed and collected for analysis.

### AMPK activity assay

After treatment, cells or tissues were lysed, and AMPK activity was measured according to the protocol of the kit (EnzyFluo AMPK Phosphorylation Assay Kit (#EAMPK-100) (BioAssay Systems, Hayward, CA, USA)). Briefly, the samples were added to a plate coated with an AMPK substrate, and the reactions were initiated by adding Mg^2+^ and ATP. After incubation and washing, a monoclonal antibody specific for the phosphorylated form of the substrate was added. After another round of incubation and washing, the HRP-conjugated secondary antibody was added to the wells. The color was developed using a chromogenic substrate, and the signals were measured densitometrically at 450 nm. After subtracting the OD450 of the control samples, the OD450 values of the samples were used as the relative AMPK activity values. Each experiment was performed in triplicate.

### CEBPB and P300/CBP activity assays

Evaluation of the activity of CEBPB and p300/CBP, the other targets of salicylate, was performed on cells after treatment according to the manufacturer's protocol using the following kits: C/EBP-beta (Phospho-Thr235/188), Colorimetric Cell-Based ELISA Kit (AssayGenie, Dublin, Ireland), and P300/CBP Colorimetric Cell-Based ELISA Kit (Boster, Pleasanton, CA, USA). Relative values were determined based on the absorbance at 595 nm when the concentration of salicylate was 0 mM. Each experiment was performed in triplicate.

### Cell proliferation assay

To evaluate cell division ability, BrdU uptake was assessed using the Frontier BrdU Cell Proliferation Assay kit (ExAlpha Biologicals, Shirley, MA, USA) according to the manufacturer's protocol. Briefly, salicylic acid solutions of various concentrations (0, 0.1, 0.25, 0.5, 1, and 5 mM in 100 μL of PBS) were administered to the treated groups in the growth medium, whereas the control group received the same amount of PBS (100 μL). After 12 h, the medium was replaced, and the cell proliferative capacity was assessed using a kit. Absorbance was measured at 450/550 nm. Each experiment was performed in triplicate.

### Immunocytochemistry

The cells in each group were incubated overnight at 4 °C with Acti-stain 488 phalloidin (Thermo Fisher Scientific) and anti-E-cadherin (EP700Y; Abcam, Cambridge, UK) diluted in 1:100 PBS. After washing three times with PBS, the slides were incubated with Alexa Fluor 555-conjugated goat anti-rabbit antibody (Thermo Fisher Scientific) diluted in 1:2,000 PBS for 1 h at room temperature. After incubation, the nuclei were counterstained for visualization using a DAPI solution (Fujifilm Wako Pure Chemicals; 1:500).

### Immunohistochemistry

Tissue samples were washed three times with 0.2% Triton X-100 in PBS (PBST) for 15 min. Cells were washed once with PBS for three minutes and blocked with a 3% bovine serum albumin (BSA)/PBS solution for 1 h at room temperature. For actin staining, cells were incubated overnight at 4 °C with Acti-stain 488 phalloidin (PHDG1-A; Cytoskeleton, Inc., Denver, CO, USA; 1:140). After washing three times with PBST, the nuclei were incubated with DAPI solution (Fujifilm Wako Pure Chemicals; 1:500) and mounted on glass slides using ProLong Gold (Thermo Fisher Scientific).

For immunostaining against other antigens, after blocking, the primary antibody was diluted in blocking solution (3% BSA/PBS) and the tissue was incubated overnight at 4 °C with rat anti-E-cadherin antibody diluted in PBS (SAB4200684; Sigma-Aldrich, St. Louis, MO, USA; 1:500). Samples were then washed three times with PBST and incubated for 1 h with Hoechst 33,342 (1:500) and Alexa Fluor secondary antibodies (ab150158; goat anti-rat IgG Alexa Fluor 555; 1:200; Invitrogen, Waltham, MA, USA). After washing twice with PBST and once with PBS, the cells were mounted on glass slides using ProLong Gold (Invitrogen). All slides were viewed under a confocal laser-scanning microscope (FLUO-VIEW FV3000).

### Laser microdissection (LMD)

LMD was performed using a PALM MicroBeam (Carl Zeiss, Germany). The manufacturer’s recommended slides and collection tubes (AdhesiveCap 500 opaque; Carl Zeiss) were used. After adjusting the aperture and intensity using a 20 × magnification objective lens, the tissue was carefully cut, and samples from the wound margins were collected. Tube caps were filled with Buffer RLT (RNeasy Micro Kit, Qiagen, Germany) containing β-mercaptoethanol to allow for the isolation of intact RNA using an RNeasy Micro Kit (Qiagen, Hilden, Germany).

### RNA isolation and reverse transcription

Total RNA was extracted from cells or skin tissue according to the manufacturer’s instructions and mixed with SuperScript™ IV VILO™ Master Mix (Thermo Fisher Scientific) in a T100™ thermal cycler (Bio-Rad Laboratories, Inc., Hercules, CA, USA) for 5 min at 25 °C, 10 min at 55 °C, and 10 min at 80 °C to inactivate reverse transcriptase and produce cDNA.

### Quantitative real-time polymerase chain reaction (RT-qPCR)

RT-qPCR was performed using an Applied Biosystems 7500 Fast Real-Time PCR System (Thermo Fisher Scientific). A total of 40 cycles were performed, and the fluorescence of each sample was measured at the end of each cycle. PCR was performed in two major steps: holding the reagents at 95 °C for 3 s (denaturation) and 60 °C for 30 s (annealing and extension). In the subsequent melting curve analysis phase, the temperature was increased from 60 to 95 °C, and fluorescence was measured continuously. Rac1 (Mm01201653_mH), Arp2 (Mm07300461_g1), and Arp3 (Mm02342769_g1) were used as primers (Thermo Fisher Scientific). The PCR Master Mix (Cat. 4,352,042; Applied Biosystems, Foster City, CA, USA) was used in accordance with the manufacturer’s instructions, and ACTB (Mm02619580_g1) was used as the control gene for normalization. Gene expression levels in normal cells or those at unwounded sites were used as the baseline and fold-change values were determined using the 2-ΔΔCt method.

### Western blotting

Total protein was extracted from cells and tissues using RIPA buffer (Santa Cruz Biotechnology, Santa Cruz, CA, USA). Each sample (30 μg) was electrophoresed on 10% polyacrylamide gels using Mini-PROTEAN® TGX™ Precast Gels (Bio-Rad Laboratories, Inc., CA, USA) and transferred to a Trans-Blot Turbo Transfer System (Bio-Rad). After blocking with 3% non-fat milk at room temperature for 1 h, the following primary antibodies were diluted in the blocking solution at 4 °C overnight: Rac1 (1:100, Abcam), ARP2 (1:200, Abcam), ARP3 (1:200, Abcam), and GAPDH (1:2000, Santa Cruz Biotechnology). The following day, the samples were incubated with donkey anti-goat IgG H&L (HRP) (ab6885; Abcam) and goat anti-rabbit IgG H&L (HRP) (ab205718; Abcam) for 1 h at 37 °C. After washing, the immunoreactive protein bands were visualized using an electrochemiluminescence detection kit (Pierce Biotechnology, Rockford, IL, USA), and a chemiluminescence imager (ImageQuant LAS4000 mini; GE Healthcare, Chicago, IL, USA) was used to image the bands. Image analysis was performed using the ImageJ software version 1.53p (NIH, Bethesda, MD, USA). Each experiment was performed in triplicate.

### Modified manchester scar scale (MSS)

Wound evaluation was performed using a modified MSS adapted to the skin properties of mice using a previously described method^19^. The histological technique involved division of the skin into the epidermis and dermis, and the dermis was further divided into the papillary and reticular dermis; evaluation was subsequently performed using Masson’s trichrome staining (Table S1). Histological evaluation is normally scored on a scale of 0–32 points, with lower histological scores indicating better results. However, in this study, the following considerations were made: (1) a score of 5 was not used because the mice had no keloid tissue; (2) because the papillary and reticular layers of the dermis were indistinguishable, the evaluation was performed on the dermis without distinction; and (3) because there were no changes in the epidermis of all wounds, the epidermal score was converted to 0. The Thee scoring scale was adjusted from 0 to 12 based on these changes.

### Statistical analysis

Data are expressed as the mean ± standard error of the mean, and statistically significant differences between the two groups were determined using Statistica software (version 9.0; StatSoft, Tulsa, OK, USA). Differences were considered statistically significant at *P* < 0.05. The Mann–Whitney *U* test was used to analyze the differences between the two groups.

## Results

### Salicylate promotes regeneration of mouse fetus skin through AMPK activation and actin cable formation

**Figure 1:**
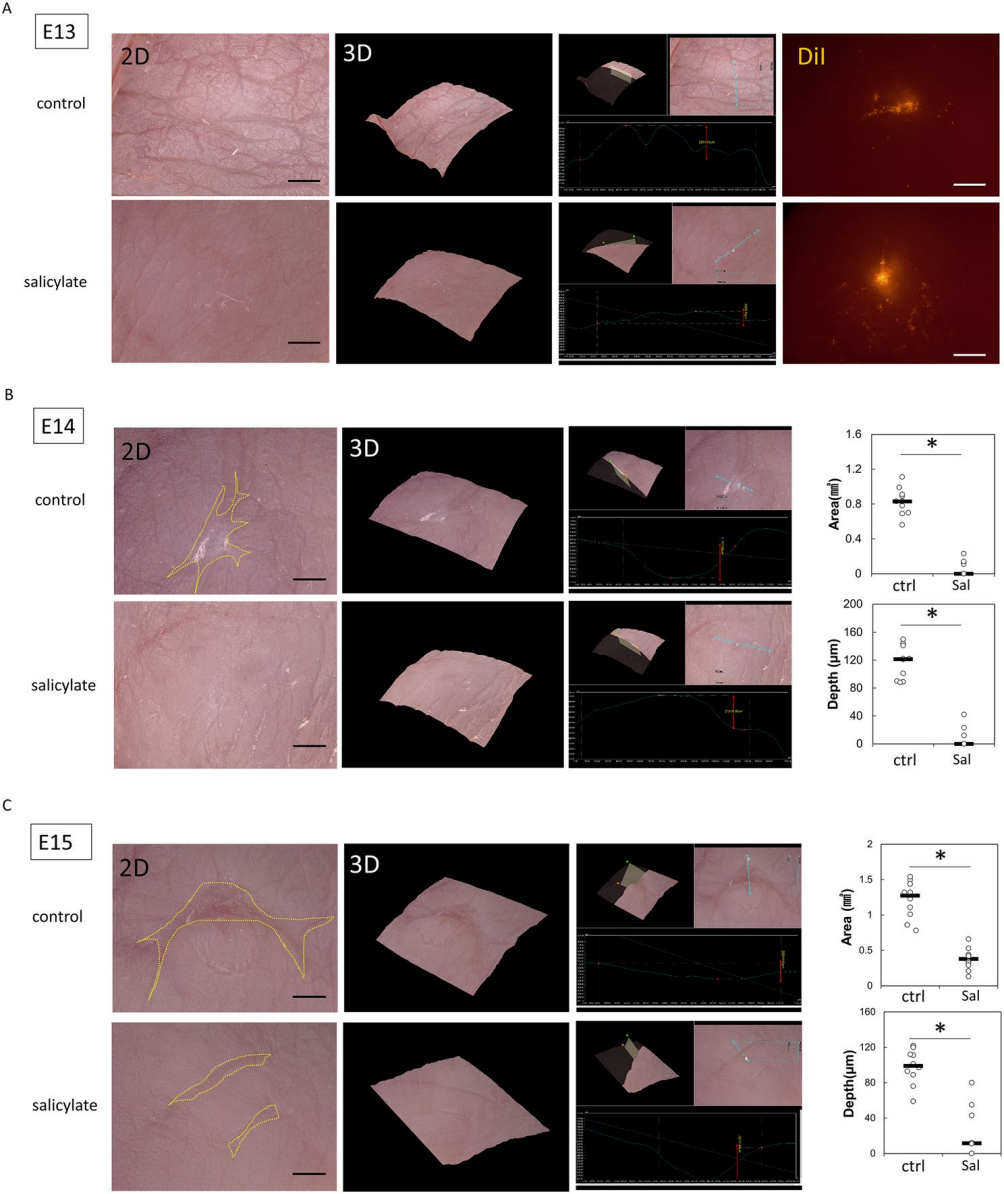

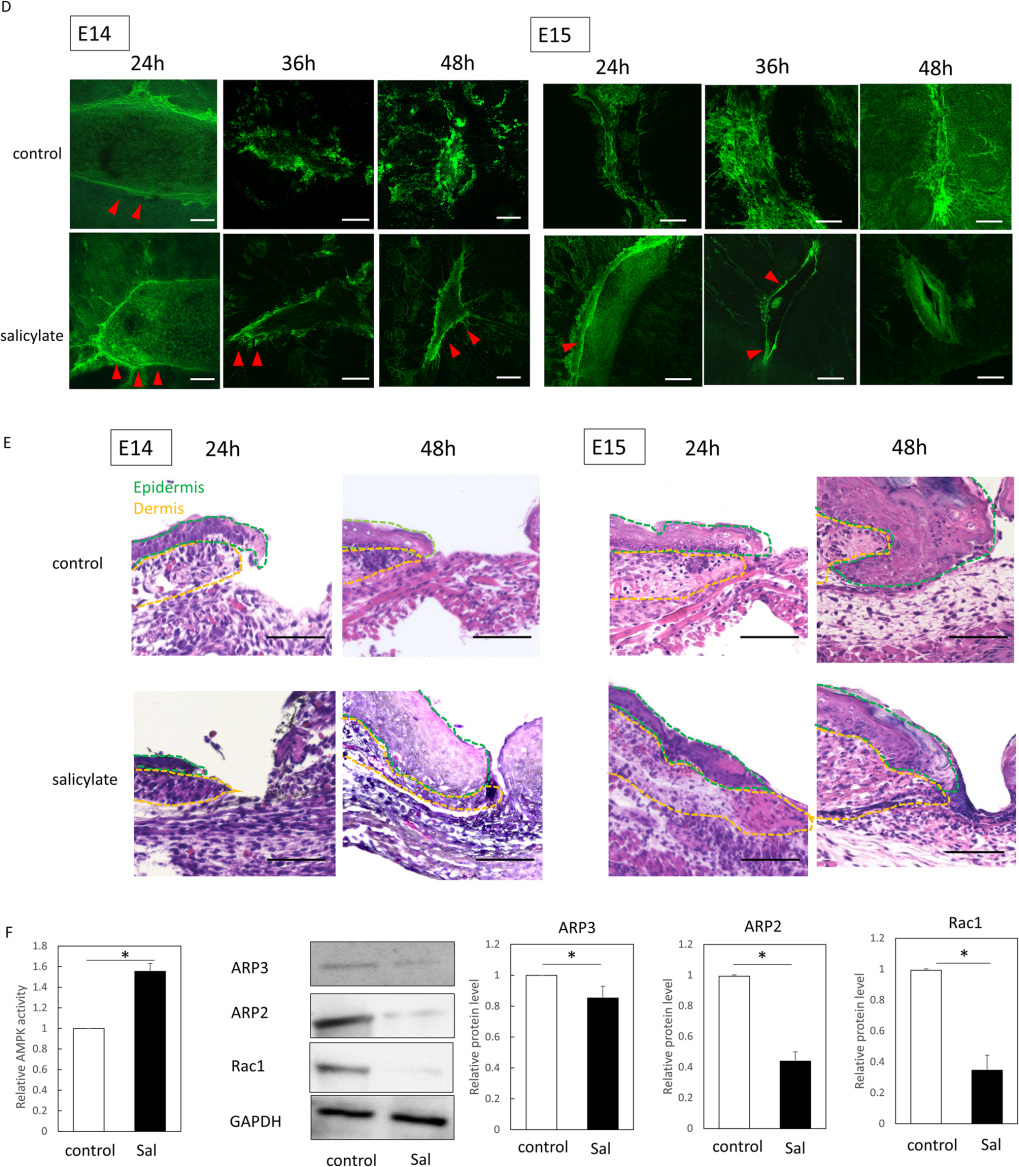

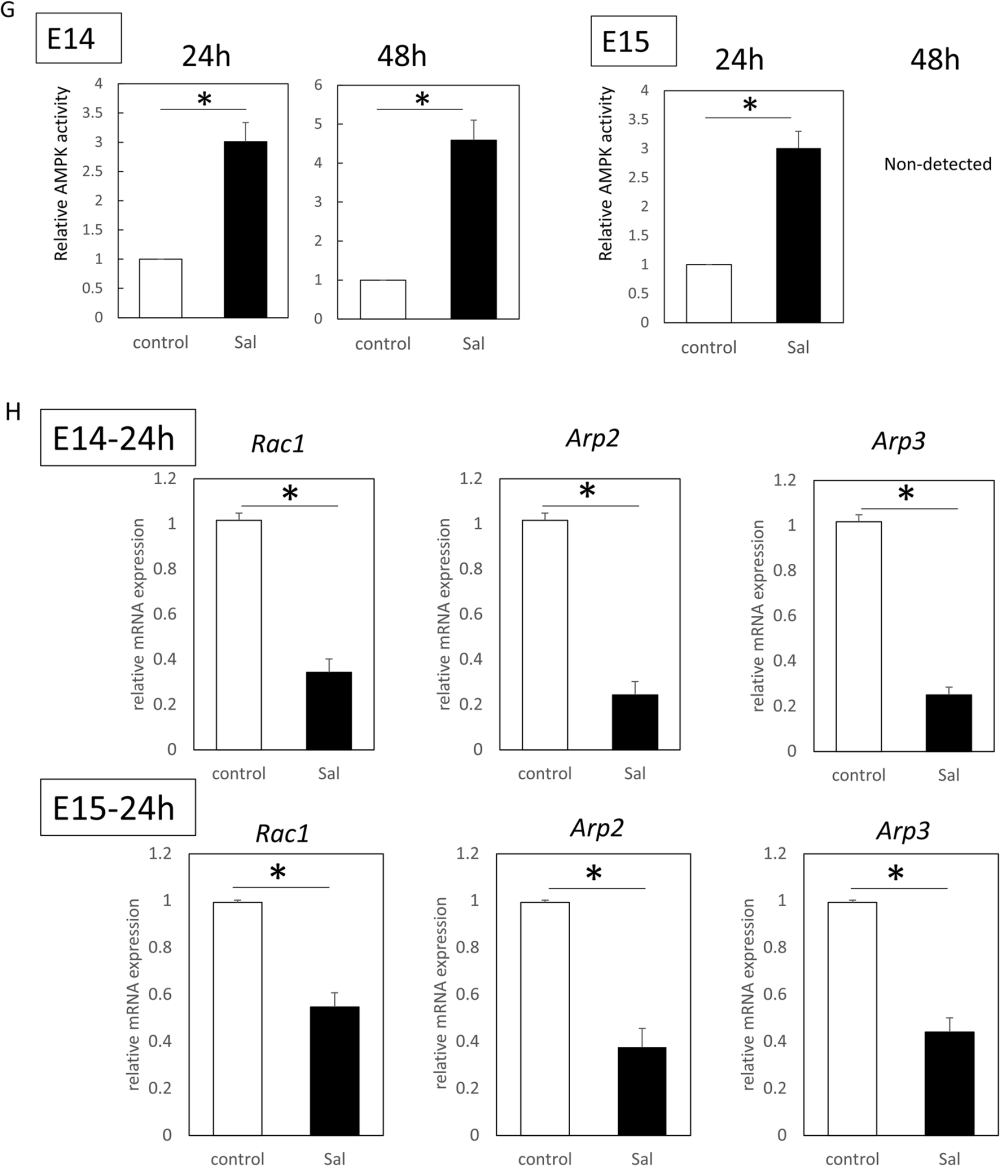
Effects of salicylate on fetal mouse wound healing. (**a)** Evaluation of wounds following salicylate administration on E13. With or without administration, the wound completely regenerated without visible marks. Orange: Location where the wound was created (DiI fluorescent dye was incorporated into the skin). Bar = 500 µm. (**b)** Evaluation of wounds following salicylate administration on E14. The skin of mice in the salicylate-treated group regenerated completely after 72 h of salicylate administration, whereas that of the control group left visible scars and showed disruption of skin texture. Yellow dashed line: extent of visible marks. Bar = 500 µm. (**c**) Evaluation of wounds following salicylate administration at E15. Salicylate administration on E15 reduced visible scars after 72 h, with a significant reduction in depth and area. Yellow dashed line; extent of visible marks. Bar = 500 µm. (**d**) Actin cable formation during the wound healing process on E14 and E15. In E14, actin cables, which disappear 36 h after injury, are formed during the entire wound healing process by salicylate administration; in E15, actin cables do not form but are formed up to 36 h after salicylate administration. Red arrows indicate areas of actin cable formation. Bar = 20 µm. (**e**) Positional relationship of the epidermis dermis during the wound healing process on E14 and E15 (hematoxylin–eosin staining). In E14, the epidermis contacts the fascia after 48 h in controls, but salicylate treatment keeps the epidermis and dermis in position; in E15, the dermis covers the wound before the epidermis until at least 24 h after salicylate treatment. Orange line: epidermis; green line: dermis; bar = 100 µm. (**f**) Histological analysis of wounds 72 h after injury on E14 and E15. The panniculus carnosus muscle was repaired and contracted at the base of the wound following salicylate administration at both E14 and E15. Yellow dashed line: panniculus carnosus muscle. Bar = 200 µm. (**g)** AMPK activation at the wound margin induced by salicylate administration. Salicylate administration significantly activated AMPK in the wound for up to 48 h after E14 and 24 h after E15. (**h**) Evaluation of AMPK signaling-related genes following salicylate administration. Salicylate treatment significantly decreased the expression of all genes. *ctrl* Control, *Sal* Salicylate. *; *P* < 0.05.

To investigate the effects of salicylate on the transition between skin regeneration and healing in fetal mouse skin, salicylate was administered following wounding, and the formation of actin cables in the wound was analyzed. At E13, the wound regenerated completely without a visible mark with or without salicylate administration (Fig. 1A). The 2D image shows that the texture was visually regenerated. In addition, visible scars were recognized as depressions separated from the texture in the 3D reconstructed image, whereas the texture was observed as small ridges in the regenerated image. The creation of wounds in the regenerating areas was determined using the fluorescent dye DiI, which can be observed in the skin. However, on E14, the skin of the mice in the salicylate group regenerated completely after 72 h of salicylate administration, whereas that of the control group left visible scars and showed disruption of the skin texture (Fig. 1B). Salicylate administration at E15 reduced visible scars after 72 h, with significant reductions in depth (*P* = 0.000021) and area (*P* = 0.000017) (Fig. 1C). These changes were not observed when the concentration of salicylate was reduced to 0.1 mM, and skin regeneration was not observed at E14 or scar reduction at E15 (Supplemental Figure S1A). Alternatively, when salicylate was administered at a higher concentration (100 mM), the wound was ulcerated without epithelialization (Supplemental figure S1B).

Observation of actin dynamics in the wound revealed cables that could be observed on E14 48 h after wounding. On E15, when actin cables were not formed during the wound-healing process, salicylate administration resulted in the formation of cables that were present for up to 36 h (Fig. 1D).

During the wound-healing process, the embryonic pattern of skin regeneration is characterized by the maintenance of epidermal and dermal positioning. In the control group, the epidermis overcame the dermis and contacted the fascia on both E14 and E15; however, salicylate administration resulted in epithelialization and maintenance of the epidermal–dermal positional relationship (Fig. 1E). In addition, observations of the post-healing wound tissue revealed that another element involved in skin healing, the panniculus carnosus muscle, was repaired and contracted at the base of the wound following salicylate administration at both E14 and E15 (Fig. 1F).

AMPK activity was significantly enhanced in the salicylate-treated group compared to the control at E14 (24 h, *P* = 0.00019; 48 h, *P* = 0.00017) and 15 (24 h, *P* = 0.00031) (Fig. 1G). Furthermore, the expression levels of Rac1 (E14, *P* = 0.0018; E15, *P* = 0.00033), ARP2 (E14, *P* = 0.0019; E15, *P* = 0.000021), and ARP3 (E14, *P* = 0.000023; E15, *P* = 0.00033), which are downstream signals of AMPK, decreased, indicating AMPK activation (Fig. 1H).

### *Salicylate specifically targets only AMPK, and alters migration ability *via* actin remodeling of keratinocytes through regulation downstream of Rac1 signaling*

**Figure 2:**
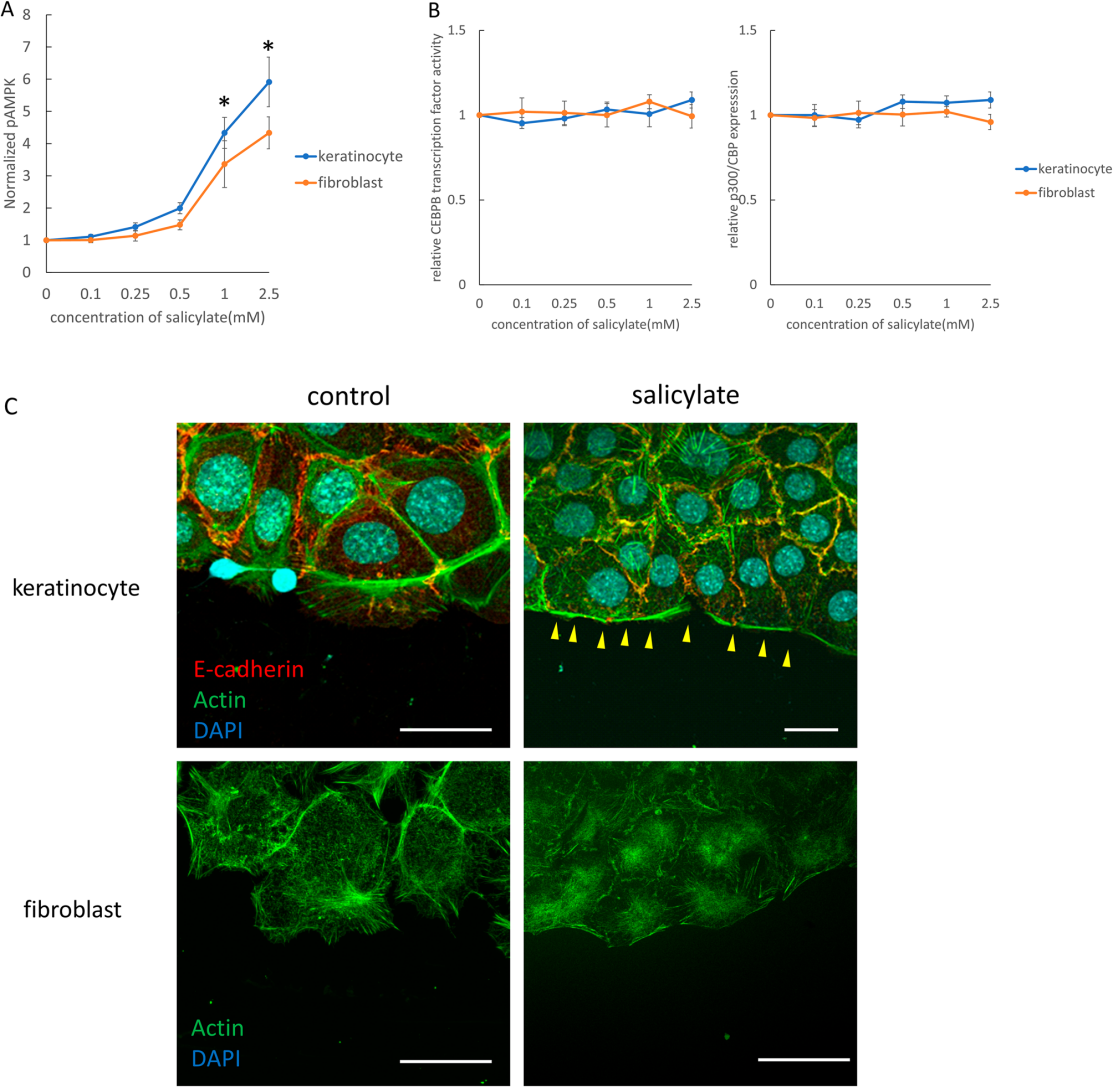

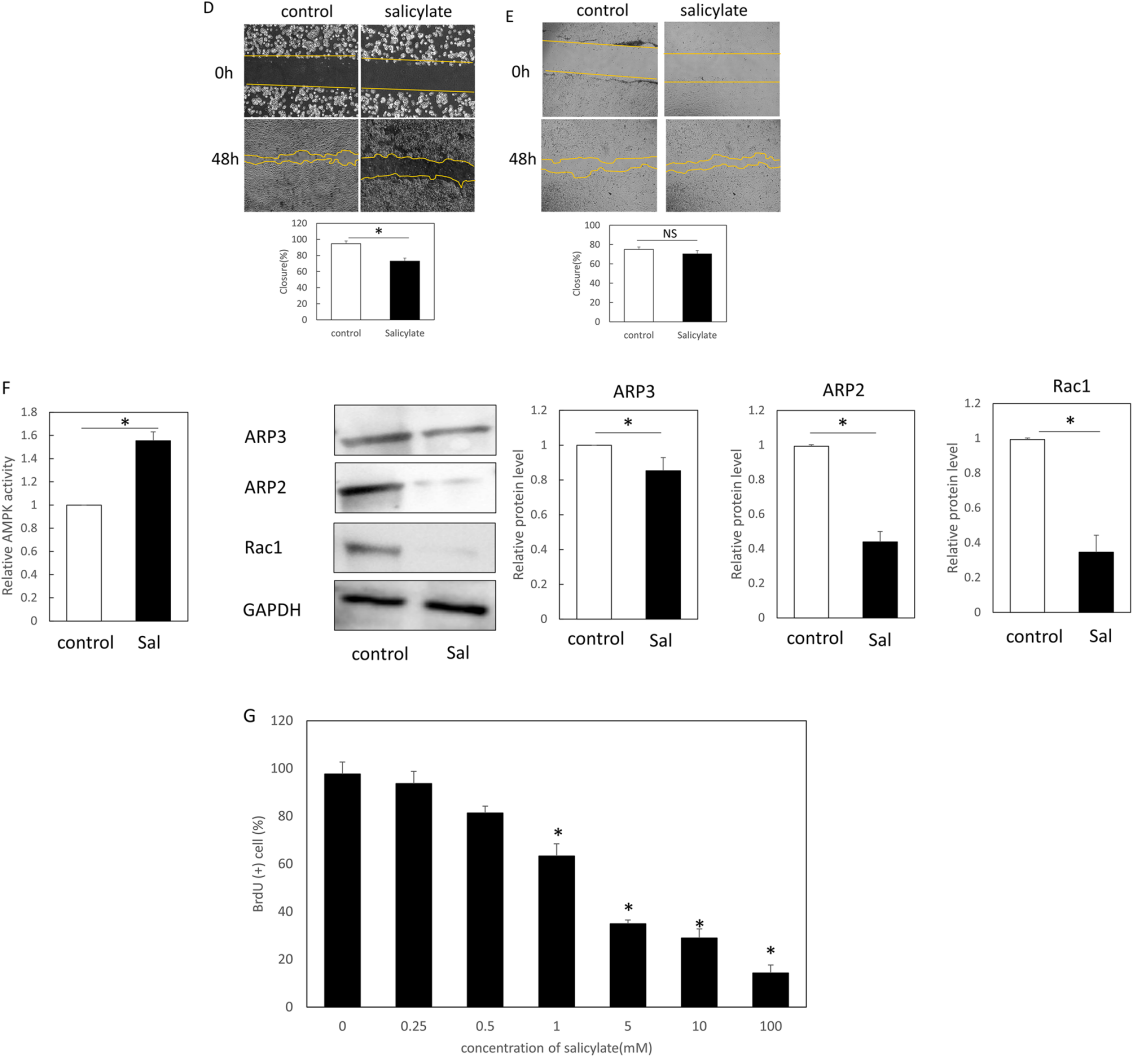
Evaluation of the effects of salicylate on mouse skin cells in vitro. (**a)** Evaluation of the dose-dependent AMPK activation of salicylate in mouse cells. AMPK was significantly activated by salicylate concentrations above 1 mM compared to the controls. (**b**) Evaluation of the activity of other target molecules of salicylates, CEBPB, and p300/CPB. The activity of these molecules was not altered by salicylate administration. (**c**) Actin dynamics following salicylate treatment in keratinocytes and fibroblasts. In keratinocytes, salicylate administration affected stress fiber formation and filopodia formation, but not in fibroblasts. Yellow arrows indicate points where cable-like structures were formed on the cell surface at the edges of the scratch (wound). Green: actin (phalloidin), red: E-cadherin, and blue: DAPI. Bar = 20 µm. (**d**) Changes in the migration capacity of keratinocytes in the scratch assay following salicylate treatment. Administration of salicylate reduced the migratory ability of keratinocytes. Orange lines indicate wound margins. (**e**) Changes in fibroblast migration in the scratch assay following salicylate treatment. Salicylate administration did not affect the migratory capacity of fibroblasts. Orange lines indicate wound margins. (**f**) Evaluation of AMPK signaling activity in keratinocytes following salicylate treatment. Salicylate treatment increased AMPK activity and decreased ARP2, ARP3, and Rac1 expression in keratinocytes. (**g**) Effect of salicylate on the proliferative ability of keratinocytes (BrdU assay). Administration of salicylate above 1 mM significantly reduced the ability of keratinocytes to proliferate. *ctrl* Control, *Sal* Salicylate. *; *P* < 0.05.

To examine the effects on mouse keratinocytes and fibroblasts, salicylate was administered to each cell at varying concentrations, and significantly increased AMPK activity (phosphorylation) was observed compared to the control (non-treated) group at concentrations above 1 mM (1 mM, *P* = 0.021; 2.5 mM, *P* = 0.0034) (Fig. 2A). To confirm the specificity of the AMPK-promoting activity of salicylate, we evaluated the activity of other targets, CEBPB and p300/CPB, and found no significant activation of either molecule at a similar concentration range (Fig. 2B). Thus, we propose that salicylate may induce actin remodeling in the epidermis. Therefore, we performed a scratch assay with mouse keratinocytes in vitro. The results of this assay showed that cells at the wound margin formed actin-induced filopodia in the control group, whereas the salicylate-treated group maintained the stress fiber morphology as an intracellular skeleton and formed a cable-like structure at the wound margin (Fig. 2C). In contrast, in the scratch assay using fibroblasts, the control group showed formation of filopodia at the wound margin, as in keratinocytes, while the salicylate-treated group showed no cable formation but suppressed the formation of filopodia and slightly formed intracellular stress fibers (Fig. 2C).

The migration abilities of keratinocytes and fibroblasts were significantly decreased by salicylate treatment (*P* = 0.011) (Fig. 2D). However, no significant difference was observed in fibroblasts, although migration tended to decrease (*P* = 0.061) (Fig. 2E). In addition, salicylic acid-treated keratinocytes showed increased AMPK activity (*P* = 0.00021) and decreased ARP2 (*P* = 0.00019), ARP3 (*P* = 0.00018), and Rac1 (*P* = 0.00027) expression (Fig. 2F).

The effect of salicylic acid on the mitogenic potential of keratinocytes was also examined using the BrdU assay, which showed that cell division was significantly inhibited at concentrations above 0.5 mM (0.5 mM, *P* = 0.021; 1 mM, *P* = 0.00021; 2.5 mM, *P* = 0.000019) (Fig. 2G).

### Salicylate promotes wound healing in adult mice

**Figure 3:**
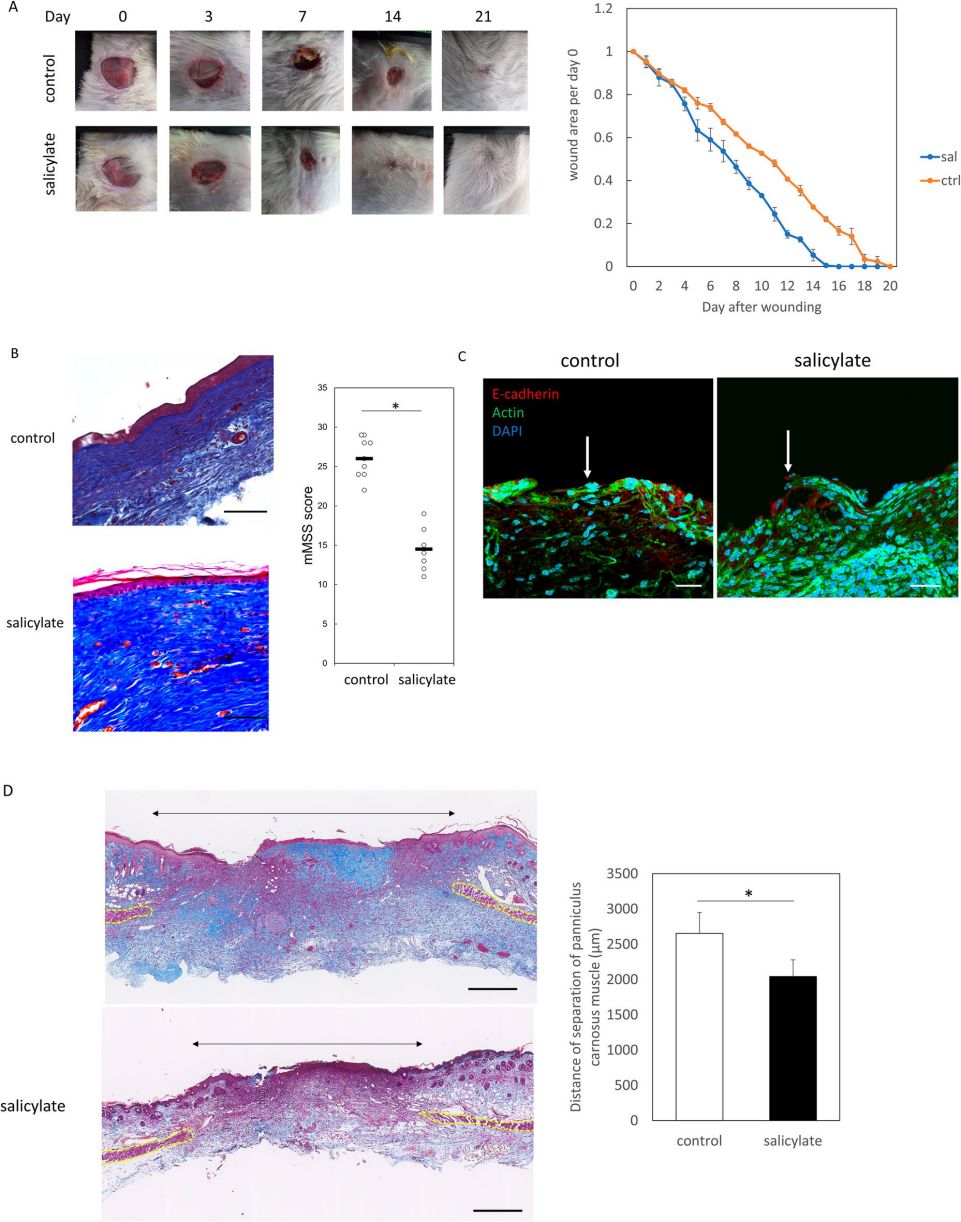

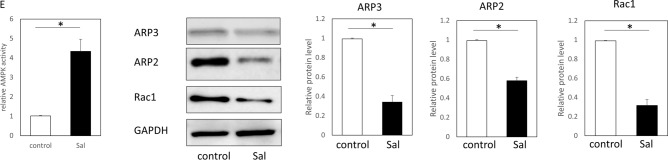
Effects of salicylate administration on wound healing in adult mice. (**a**) Comparison of epithelialization speed after salicylate administration. Administration of salicylate promoted wound closure. (**b)** Assessment of wound scarring (Masson trichrome staining). Bar = 200 µm. mMSS: modified Manchester Scar Scale. The degree of fibrosis reduced in the treatment group. *P < 0.05. (**c**) Observation of actin dynamics in the wound. White arrows indicate wound edges. Salicylate treatment resulted in wound margin-specific expression of E-cadherin (red) and altered the expression pattern of actin (green). Bar = 20 µm. (**d**) Evaluation of panniculus carnosus muscle contraction in adult mice following salicylate administration (Masson trichrome staining). The administration of salicylate was also found to promote repair and reduce tissue gaps in wounds. Yellow dashed line: panniculus carnosus muscle. Black arrows: gaps between dermatoglyphics (measurement area). Bar = 500 µm.*P < 0.05. (**e**) Evaluation of AMPK signaling-related genes in the wound margin following salicylate treatment. Administration of salicylate also activated AMPK and decreased the expression of ARP2, ARP3, and Rac1 in adult mouse wounds. *ctrl* Control, *Sal* Salicylate.

To investigate whether the effects of salicylate on promoting wound healing were also present in adult mice, salicylate was administered daily to wounds, resulting in accelerated epithelialization (Fig. 3A). Scars were also scored, and the results showed that the degree of fibrosis was reduced in the treated group (*P* = 0.0021) (Fig. 3B). Histologically, E-cadherin, which tethers F-actin, was expressed at the wound margin, although there was no actin cable formation in adult mice, and the morphology of actin expression at the wound margin was altered (Fig. 3C). In the panniculus carnosus muscle, salicylate administration also promoted repair and reduced tissue gaps in wounds (*P* = 0.033) (Fig. 3D). Furthermore, we found that AMPK activity was upregulated (*P* = 0.00011), and the expression of Rac1 (*P* = 0.0018), Arp2 (*P* = 0.00027), and Arp3 (*P* = 0.00022) was downregulated in wounds (Fig. 3E), which is similar to the results observed in fetuses.

## Discussion

Complete wound regeneration preserves the structure and physiological function of the skin, including stimulus sensation and perception, whereas incomplete wound regeneration leads to fibrosis and scarring. Adult mammals cannot regenerate their skin after injury. However, newts and African clawed frogs, which can regenerate wounds without fibrosis, as well as fetuses, are good models for developing skin-regenerating treatments^20–22^. However, several aspects of skin regeneration mechanisms remain unexplored. We have shown that between E13 and E14, the formation of actin cables and the positions of the epidermis and dermis are the primary factors involved in regeneration^10,20^.

In this study, we hypothesized that when the skin cannot regenerate, actin is mobilized to form filopodia for cell migration rather than cable formation. Hence, we investigated whether wound healing occurred through the formation of embryonic-type actin cables by regulating actin dynamics. On E14, there should have been scarring; however, salicylate treatment induced actin cable formation and skin regeneration. Additionally, the dermis contracted before the epidermis, which was consistent with that observed at E13. Furthermore, we observed that the panniculus muscle showed accelerated repair of the wound gap, although it did not completely regenerate. To elucidate the mechanism underlying this observation, salicylate was administered to mouse keratinocytes and fibroblasts, which are two important cell components in the skin. Subsequent analysis showed that AMPK in keratinocytes was significantly activated at 1 mM, whereas the other targets (CEBPB and p300/CPB) were not activated in the same concentration range, confirming the concentration and cell specificity of salicylate. Furthermore, salicylate treatment induced actin cable formation at keratinocyte wound margins in vitro and inhibited filopodia formation in keratinocytes. This resulted in a decrease in cell migration capacity. Fibroblasts showed no obvious cable formation or significant changes in their migration capacity. In adult mice, salicylate administration also accelerated wound closure, reduced scarring, histologically altered actin dynamics in the epidermis of wound breaks, and promoted the repair of the panniculus carnosus muscle.

Salicylate is used to replace aspirin in many medical applications^23^. Salicylate can also be administered as salsalate, which has been identified as a promising treatment for insulin resistance and type 2 diabetes^24^. Salsalate inhibits cyclooxygenase and the biosynthesis of prostanoids and protein kinase IκB kinase β in the NF-κB pathway^25,26^. Additionally, its effects on AMPK activation have only recently been elucidated, with recent research showing that it activates AMPK by binding to the AMPK β1 subunit^16,27^. Vasodilator-stimulated phosphoprotein, an actin regulatory protein, is modulated by AMPK-dependent phosphorylation, which impairs actin cytoskeleton assembly in endothelial cells^28^. This suggests that the downstream signaling pathway of AMPK is linked to various signaling mechanisms that regulate the actin cytoskeleton and that it plays different roles depending on the system or cell type.

Salicylate has multiple pharmacological targets other than AMPK^29,30^. Thus, it is very interesting to note that under our administration conditions, salicylate specifically activated only AMPK in keratinocytes and regulated Rac1 downstream signaling, thereby altering actin dynamics and downregulating stress fiber formation rather than filopodia formation. While AMPK activation inhibits mTOR-mediated keratinocyte proliferation following skin injury or stress^31^, we found that migration, another factor in epithelialization morphology, was also altered by AMPK activation. The effect of AMPK activation on actin reorganization in epithelial cells is a known phenomenon^9^; however, the fact that this affects Rac1 signaling involved in cell migration contributes to the understanding of wound-healing mechanisms^32^.

Wound closure comprises two main steps: epithelialization, which involves migration of the epidermis and dermis, and contraction, which is caused by collagen fiber formation in the dermis and constriction of the muscles and fascia^33^. The fact that wound closure was accelerated in our study despite the reduced migratory capacity of keratinocytes due to actin remodeling via AMPK-specific activation of salicylate also indicates the influence of salicylate on contraction. The fact that high concentrations of salicylate caused ulceration was consistent with the results of the inhibition of migration and cell proliferation in vitro. Remodeling of the actin cytoskeleton with the inhibition of filopodia and possibly lamellipodia is consistent with previous results showing that aspirin and salicylate inhibit TGF-β1-induced EMT in A549 lung cancer cells^34^, colon cancer cells^35^, and human lens epithelial cells^36^, and salicylates can inhibit epithelial cells at the leading edge of the epithelium.

In the dermis, AMPK activation under the same conditions did not result in obvious differences in actin dynamics or migration capacity. It is possible that wound closure with the epidermis and dermis in position may have approached embryonic-type wound healing, but the changes in myofibroblast and actin dynamics, which are involved in dermal repair, remain unclear. Actin dynamics during dermal healing are likely to differ from those in the epidermis, and changes in salicylate and AMPK activity in different subsets of fibroblasts during wound healing should be investigated in the future.

The panniculus carnosus muscle, an organ present in rodents that corresponds to the shallow fascia in humans, is also involved in contraction^37^. It is known that development actually occurs at a time point after E13, which we selected^38^. Given the existing findings that AMPK activity regulates muscle glucose utilization^39,40^ and that knockout of AMPK reduces muscle activity^41^, the effects we observed on wound healing in vivo and panniculus carnosus muscle contractility improvement may also influence this phenomenon. The panniculus carnosus muscle promotes collagen secretion and cell viability and provides a low-resistance surface between fascial layers^42^. Alternatively, changes in the tensile strength of the wound due to contraction of the transected panniculus carnosus muscle may also play an important role in regeneration^43^. However, the underlying mechanism is not clear, and the effect of AMPK activity following salicylate administration observed in this study needs to be investigated in the future. Alternatively, it would be useful to perform experiments that separate epithelialization and contraction using other in vivo models to better define the effects of salicylate administration on wound healing^44,45^.

Another important discussion on the mechanism by which salicylate induces fetal-type skin regeneration is the behavior of TGF-β isoforms in wounds. Among the major factors contributing to the scarless repair of skin wounds during fetal development, TGF-β3 is predominant at the wound site, whereas the levels of TGF-β1 and -2 are higher in the adult wound bed owing to their release from platelets^46,47^. Thus, it has been shown on numerous occasions that suppression of TGF beta 1 and 2 in keratinocytes and fibroblasts results in scarless repair, and application of TGF beta 3 produces the same result^48–51^. Furthermore, aspirin and salicylate have been shown to suppress TGF-β1 signaling and TGF-β1-mediated fibrosis^52,53^. Therefore, the present experiments suggest that salicylate may have contributed to regeneration and fibrosis inhibition via modulation of TGF-β signaling, in addition to actin cable induction and epidermal dermal positioning.

In the present study, we focused on the direct activation of AMPK by salicylate and AMPK activation by other AMPK activators. The effects of AMPK inhibition, siRNA knockdown, or constitutive activation on actin remodeling and wound healing should be investigated in the future^54^. The effects of salicylate on contraction of the dermis (myofibroblasts) and fascia involved in wound closure, as described above, may be clarified using animal models specific to these conditions. Additionally, the skin structures in mice and humans are different; therefore, additional experiments are needed to determine whether the effects of salicylate demonstrated in this study can be applied to humans or whether there may be possible side effects.

In conclusion, we found that salicylate induced skin regeneration in fetal mice and promoted wound healing in adult animals by promoting actin remodeling via activation of AMPK in keratinocytes and promotion of panniculus carnosus muscle contraction. These findings may be used to develop novel therapeutic modalities for wound healing through skin regeneration, which will help prevent scarring.

### Supplementary Information

Supplementary Figures.

Supplementary Information 1.

Supplementary Table 1.

## Data Availability

Data supporting the findings of this study are available from the corresponding author, K.K., upon reasonable request.
