# Supplementary figures and images for: Salicylate induces epithelial actin reorganization via activation of the AMP-activated protein kinase and promotes wound healing and contraction in mice
Source: Sci Rep. 2024 Jul 16;14:16442. doi: 10.1038/s41598-024-67266-5 (PMC11252334; doi:10.1038/s41598-024-67266-5)

Figure 2F  
original gel data

ARP3

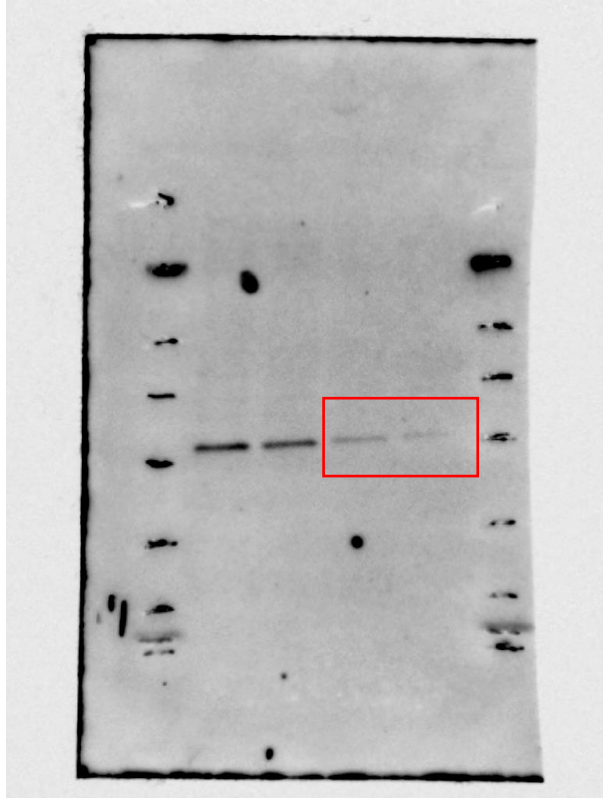

ARP2

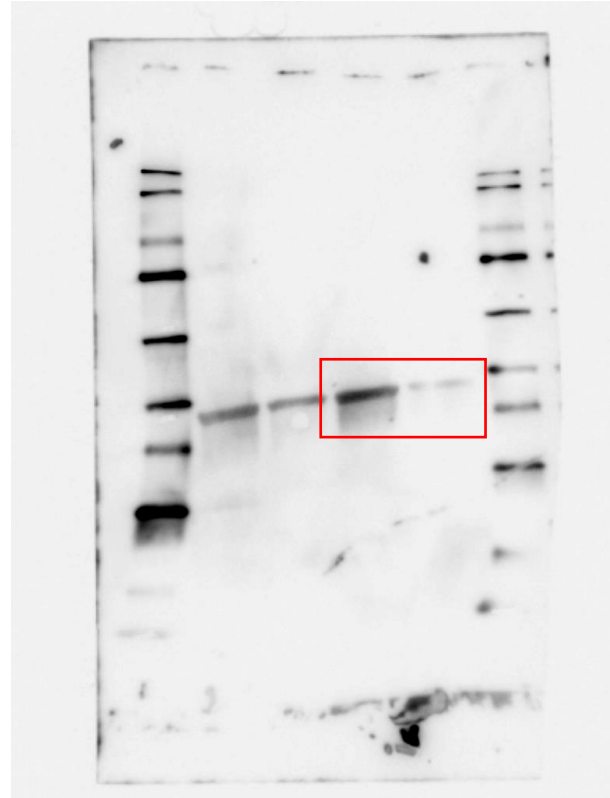

Rac1

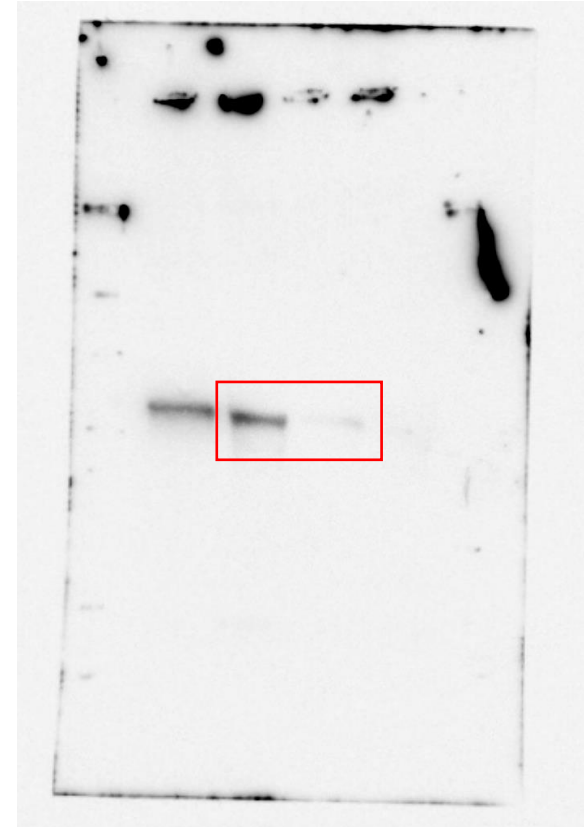

GAPDH

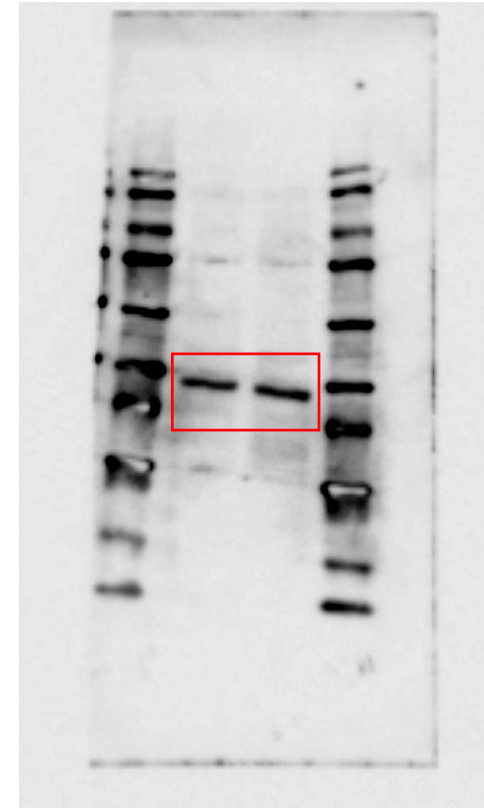

Figure 3D  
original gel data

ARP3

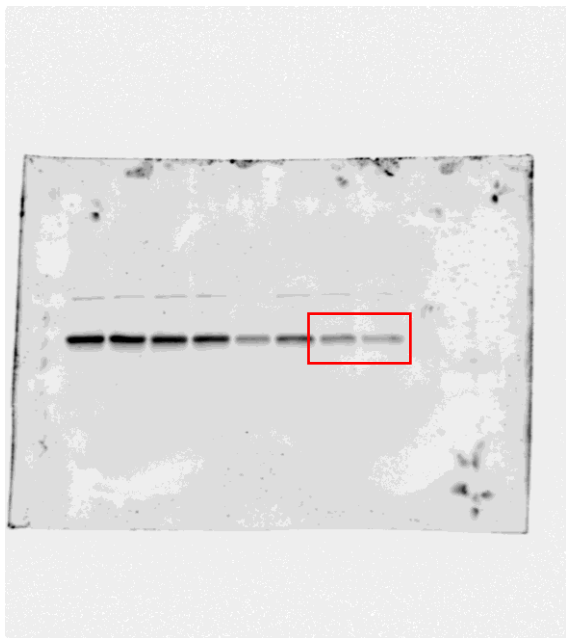

ARP2

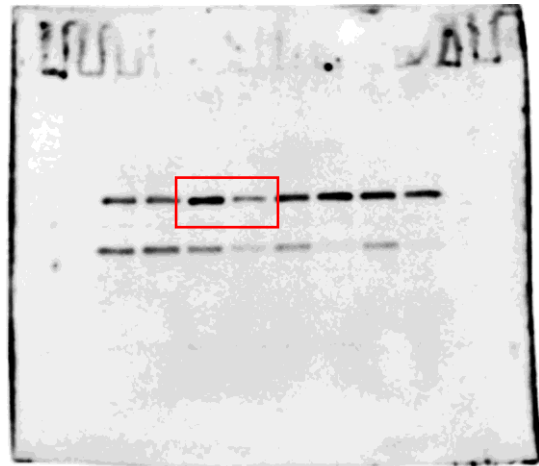

Rac1

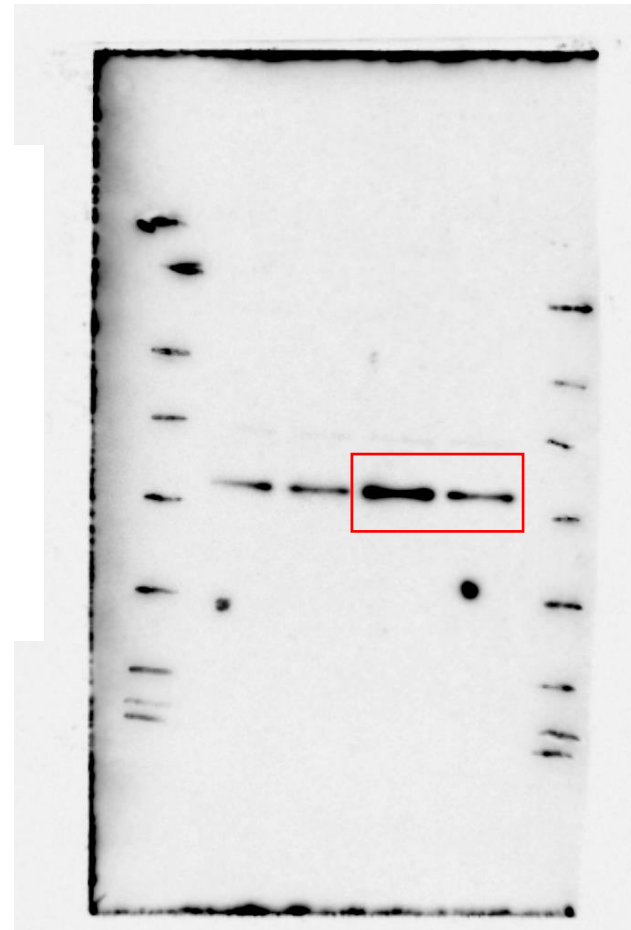

GAPDH

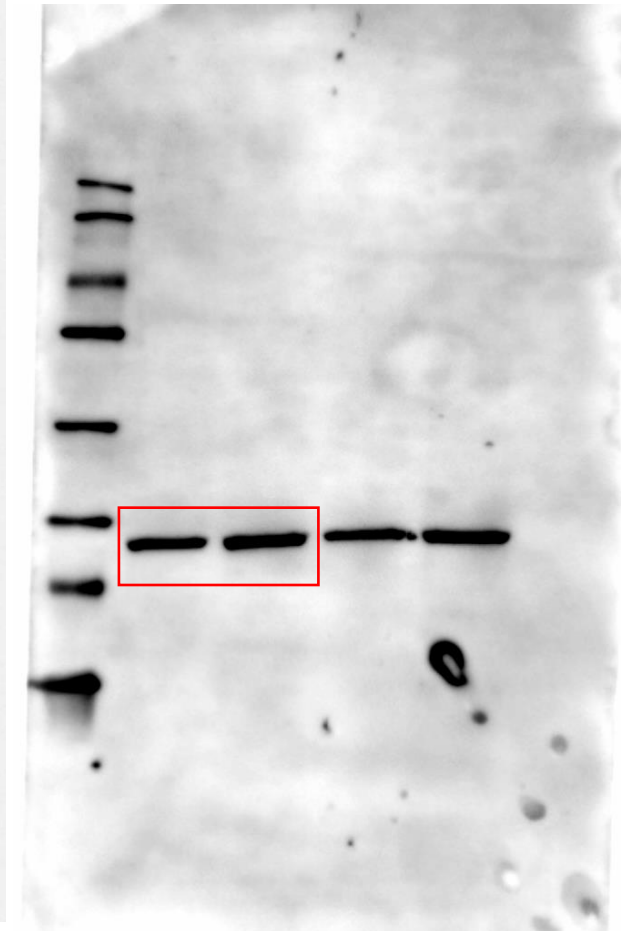

Supplement: Supplementary file 1 — Supplementary Figures. [file 41598_2024_67266_MOESM1_ESM.pdf]
