# Supplementary material for: Salicylate induces epithelial actin reorganization via activation of the AMP-activated protein kinase and promotes wound healing and contraction in mice
Source: Sci Rep. 2024 Jul 16;14:16442. doi: 10.1038/s41598-024-67266-5 (PMC11252334; doi:10.1038/s41598-024-67266-5)
Supplement: Supplementary file 2 — Supplementary Information 1. [file 41598_2024_67266_MOESM2_ESM.pdf]

S1

A

E14

E15

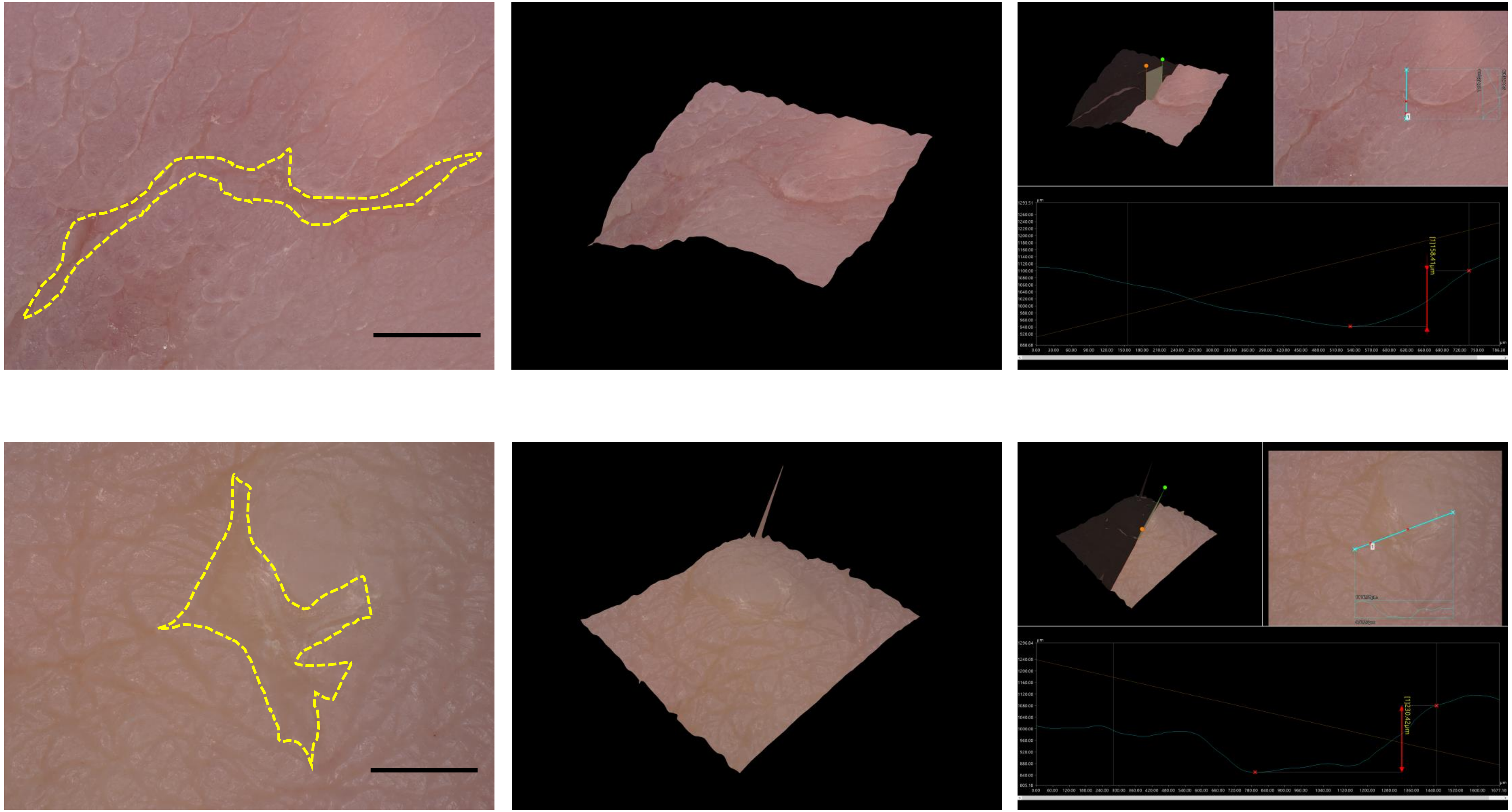

S1

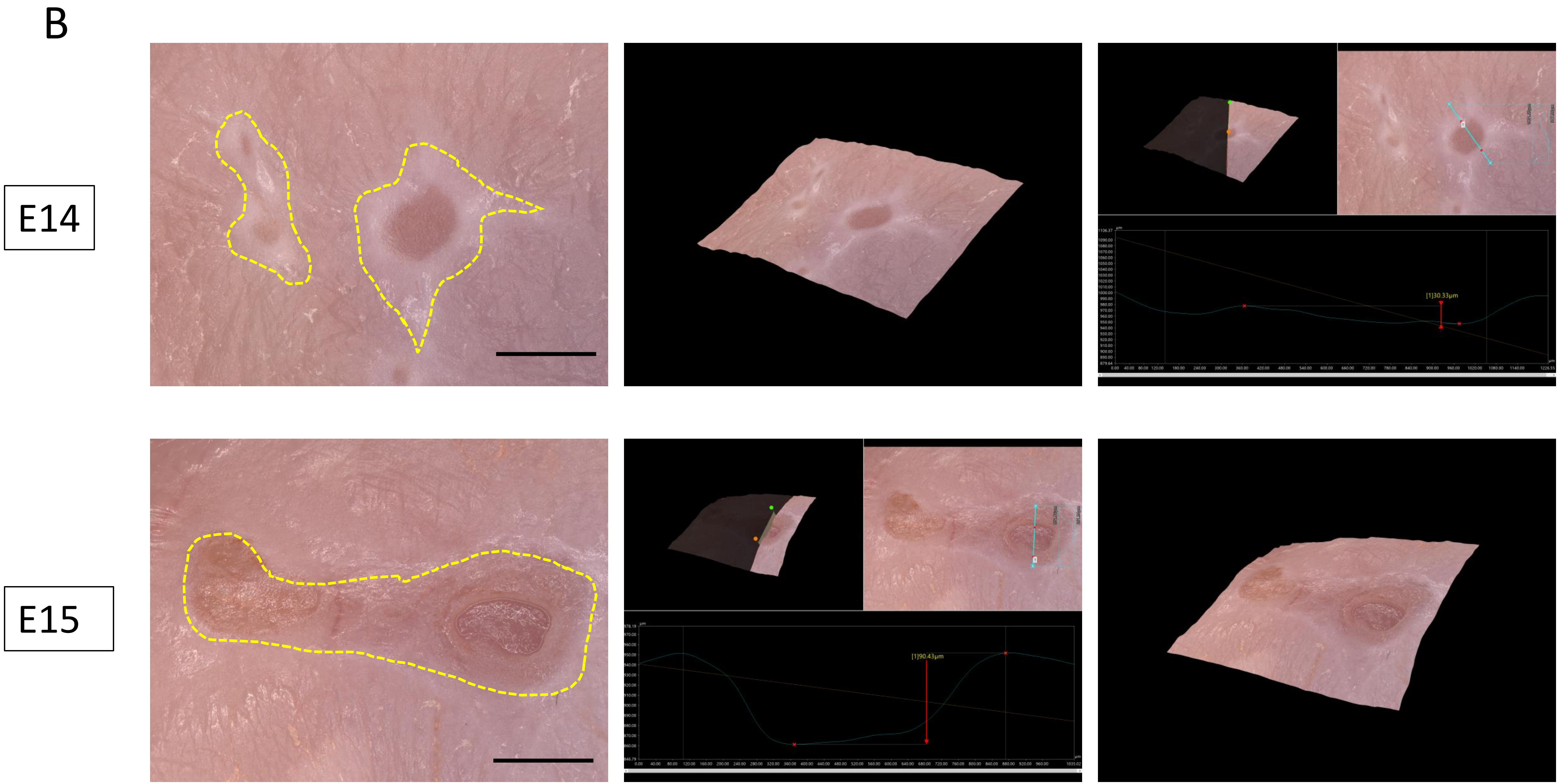

Supplement table 1

Epidermis
0 = normal
1 = some restoration of rete ridges
2 = no restoration of rete ridges
Dermis
(papillary and reticular dermis scored separately)
A.Collagen fiber orientation
0 = Normal basket-weave pattern
1 = < 25% abnormal
2 = 26 - 50% abnormal
3 = 51 - 75% abnormal
4 = 76 - 100% abnormal
5 = Keloid-like fiber orientation
B.Collagen fiber density
0 = normal fiber bundle density
1 = < 25% abnormal
2 = 26 - 50% abnormal
3 = 51 - 75% abnormal
4 = 76 - 100% abnormal
5 = Keloid-like fibers
C.Collagen fiber maturity
0 = normal fiber bundle density
1 = < 25% abnormal
2 = 26 - 50% abnormal
3 = 51 - 75% abnormal
4 = 76 - 100% abnormal
5 = Keloid-like fibers
SCORE RANGE: 0-32 (2+[5×3]+[5×3])
