# Supplementary material for: Salicylate induces epithelial actin reorganization via activation of the AMP-activated protein kinase and promotes wound healing and contraction in mice
Source: Sci Rep. 2024 Jul 16;14:16442. doi: 10.1038/s41598-024-67266-5 (PMC11252334; doi:10.1038/s41598-024-67266-5)
Supplement: Supplementary file 3 — Supplementary Table 1. [file 41598_2024_67266_MOESM3_ESM.pdf]

# Table S1

| |
|---|
| Epidermis |
| 0 = normal |
| 1 = some restoration of rete ridges |
| 2 = no restoration of rete ridges |
| Dermis |
| (papillary and reticular dermis scored separately) |
| <u>A.Collagen fiber orientation</u> |
| 0 = Normal basket-weave pattern |
| 1 = < 25% abnormal |
| 2 = 26 - 50% abnormal |
| 3 = 51 - 75% abnormal |
| 4 = 76 - 100% abnormal |
| 5 = Keloid-like fiber orientation |
| <u>B.Collagen fiber density</u> |
| 0 = normal fiber bundle density |
| 1 = < 25% abnormal |
| 2 = 26 - 50% abnormal |
| 3 = 51 - 75% abnormal |
| 4 = 76 - 100% abnormal |
| 5 = Keloid-like fibers |
| <u>C.Collagen fiber maturity</u> |
| 0 = normal fiber bundle density |
| 1 = < 25% abnormal |
| 2 = 26 - 50% abnormal |
| 3 = 51 - 75% abnormal |
| 4 = 76 - 100% abnormal |
| 5 = Keloid-like fibers |
| SCORE RANGE: 0-32 ( 2+[5×3]+[5×3] ) |
